# The impact of inflammatory cells in malignant ascites on small intestinal ICCs’ morphology and function

**DOI:** 10.1111/jcmm.12575

**Published:** 2015-06-18

**Authors:** Jing Li, Dan Kong, Yan He, Xiuli Wang, Lei Gao, Jiade Li, Meisi Yan, Duanyang Liu, Yufu Wang, Lei Zhang, Xiaoming Jin

**Affiliations:** a Department of Pathology, Basic Medical Science College, Harbin Medical UniversityHarbin, China; b Department of Gynecology, Third Affiliated Hospital of Harbin Medical UniversityHarbin, China; c Department of Pathology, Basic Medical Science College, Harbin Medical UniversityDaqing, China; d Department of Orthopedics, Second Clinical Hospital, Harbin Medical UniversityHarbin, China

**Keywords:** malignant ascites, interstitial cell of Cajal, inflammatory cells, tumour microenvironment, tumour-infiltrating lymphocyte, tumour-associated macrophage

## Abstract

Malignant ascites is one of the common complication at the late stage of abdominal cancers, which may deteriorate the environment of abdominal cavity and lead to potential damage of functional cells. Interstitial cells of Cajal (ICCs) are mesoderm-derived mesenchymal cells that function normal gastrointestinal motility. The pathological changes of ICCs or the reduced number may lead to the motility disorders of gastrointestinal tract. In this study, through analysis of malignant ascites which were obtained from cancer patients, we found that inflammatory cells, including tumour-infiltrating lymphocytes, accounted for 17.26 ± 1.31% and tumour-associated macrophages, occupied 19.06 ± 2.27% of total cells in the ascites, suggesting these inflammatory cells, in addition to tumour cells, may exert important influence on the tumour environment of abdominal cavity. We further demonstrated that the number of mice ICCs were significant decreased, as well as morphological and functional damage when ICCs were in the simulated tumour microenvironment *in vitro*. Additionally, we illustrated intestinal myoelectrical activity reduced and irregular with morphological changes of ICCs using the mice model of malignant ascites. In conclusion, our data suggested that inflammatory cells in malignant ascites may damage ICCs of the small intestine and lead to intestinal motility disorders.

## Introduction

Malignant ascites is a common complication that occurs at the late stage of malignant cancers and is associated with the characteristics of persistent, massive and recurrent tumours, which seriously threaten the quality of life of patients. Malignant ascites also changes the environment of the abdominal cavity, and thus the tumour microenvironment (TME), which exerts a profound influence on the functions of specific organs, tissues and cells 1,2. Interstitial cells of Cajal (ICCs) are mesoderm-derived mesenchymal cells that contribute to normal gastrointestinal motility. Interstitial cells of Cajal generate pacemaker potentials that drive the electrical slow waves that contribute to normal neuromuscular signalling. Additionally, ICCs are involved in mechanotransduction and establish potential gradients in the membranes of smooth muscle cells 3,4. Physicians have found that patients who have been diagnosed with malignant peritoneal metastasis or ascites formation sometimes exhibit symptoms of abdominal distension, intermittent abdominal pain, nausea and vomiting 5. A study reported that the above-mentioned symptoms were related to the damage of ICCs, such as decreased number, altered morphology and dysfunction 5,6.

The number of macrophages and lymphocytes was clearly increased in the cells of the ascites fluid. Lymphocytes in the ascites are known as tumour-infiltrating lymphocytes (TILs), which are mainly composed of CD3^+^ T cells, CD8^+^ T cells and CD4^+^ T cells 7–9. Some factors in the TME could polarize macrophages towards the tumour-associated macrophages (TAMs) phenotype. Tumour-infiltrating lymphocytes and TAMs are the important messengers between the inflammatory microenvironment of the tumour and the tumour cells, as they promote angiogenesis, lymphangiogenesis and matrix remodelling. They also suppress destructive immunity and promote tumour progression when they interact with stem cells 10–13. Thus, we wondered whether TILs and TAMs are involved in the damage of functional cells, such as intestinal ICCs, whether the cytokines originate in the TILs or TAMs and how they induce the injuries of intestinal ICCs. In this study, we aim to provide evidence that inflammatory cells from malignant ascites might have negative effect on intestinal function through inducing apoptosis and function of ICCs.

## Materials and methods

### Reagents

Stem cell factor (SCF) was purchased from ProSpec-Tany TechnoGene Ltd (Ness-Ziona, Israel). M199 medium and High Glucose DMEM medium were obtained from Hyclone (Logan, UT, USA). Anti-c-kit primary antibody was purchased from Santa Cruz Biotechnology (Dallas, TX, USA). Fluorescein isothiocyanate (FITC)-labelled secondary antibody was purchased from Vector Laboratories (Burlingame, CA, USA). FITC-labelled c-kit (CD117) antibody was purchased from SanDiego eBioscience Ltd (San Diego, CA, USA). Cell counting kit-8 (CCK8) and DAPI (4′,6-diamidino-2-phenylindole, DAPI) were purchased from Fanbo Biochemicals Co., Ltd (Beijing, China). CD8 primary antibody was purchased from Abcam (Bristol, UK). CD68 primary antibody was purchased from Santa Cruz Biotechnology. PE-labelled antimouse CD8a and APC-labelled antimouse CD68 were purchased from BioLegend (San Diego, CA, USA). CA125 antibody was purchased from Maixin Biotech Co., Ltd (Fuzhou, China).

### The source of malignant ascites

All 22 ascites samples were collected from patients diagnosed with advanced-stage serous epithelial ovarian carcinoma, after obtaining written informed consent under protocols approved by the Human Research and Ethics Committee of Harbin Medical University Cancer Hospital, Department of Obstetrics and Gynecology, during the period of September 2013 to May 2014. The malignant ascites samples were centrifuged for 10 min. at 1174 × g. The supernatants were stored at −80°C, and the precipitates were used to identify the components.

### Mouse model

All C57BL/6 mice were purchased from Biological Technology Development Co., Ltd. (Liaoning, China). All experiments were performed according to the guidelines for the care and use of animals approved by the ethics committee of Harbin Medical University. Every effort was made to minimize both the number of animals used and their suffering. The approval number is HMUIRB20140022. The mice were housed at a constant temperature of 26–28°C and a humidity of 40–60%. Food and water were sterilized using ultra-high pressures and irradiated by ultraviolet (UV) light. The sterilized cages and bedding were changed weekly. A total of 100 C57BL/6 mice (8–13 days old) of both sexes were used for the extraction of small intestinal ICCs. Thirty C57BL/6 mice were used to generate the model of malignant ascites. The mice were divided into the following two groups: the control group and the ascites group. The mice were without food and water for 12 hrs before surgery and were then anesthetized (1% pentobarbital sodium 40 mg/kg) and positioned on a cork platform. Under sterile conditions, each mouse received a 0.2-cm midline abdominal incision and was peritoneally injected with 0.2 ml of MFC cells suspension (at a concentration of 8 × 10^7^/ml saline). The site was massaged for 2 min., and the cavity was closed. After 14 and 21 days, the ascites fluid was collected from abdominal cavity and stored at −80°C, the mice were scarified.

### Cell lines

The mouse forestomach carcinoma cell line (MFC) was obtained from the Type Culture Collection of the Chinese Academy of Science (Shanghai, China). Cells were cultured with Modified Eagle’s Medium with RPMI1640 after resuscitation. The culture medium was removed from the cultures at passage 3rd and was centrifuged for 10 min. at 1500 r.p.m.; the supernatants were then collected for future use. Subsequently, cells were digested, collected and centrifuged at 1500 r.p.m. for 10 min. The supernatants were discarded, physiological saline solution was added, mixed and subsequently used to create an animal model.

The macrophage cell line RAW246.7 was also obtained from the Type Culture Collection of the Chinese Academy of Science. Cells were cultured with High Glucose DMEM medium after resuscitation. The culture medium was removed from the cultures at passage 3rd and was centrifuged for 10 min. at 1500 r.p.m.; the supernatants were collected until further use.

### Enzymatic dissociation of ICCs and cell culture

C57BL/6 mice (8–13 days old) of both sexes were anesthetized with ether and killed by cervical dislocation. The mesentery was carefully removed. The small intestine from 1 cm below the pyloric ring to the caecum was resected and opened along the mesenteric border. The luminal contents were removed by washing with Ca^2+^-free Hanks solution (containing in mM: KCl 5.36, NaCl 125, NaOH 0.34, Na_2_HCO_3_ 0.44, glucose 10, sucrose 2.9 and HEPES 11). The tissues were pinned to the base of a Sylgard dish and the mucosa was removed by sharp dissection. Small strips of intestinal muscle (that contained both circular and longitudinal muscles) were equilibrated in Ca^2+^-free Hanks solution for 30 min. The cells were then dispersed with an enzyme solution composed of collagenase Type II 1.3 mg/ml, bovine serum albumin 2 mg/ml, trypsin inhibitor 2 mg/ml and ATP 0.27 mg/ml at 35°C for 12 min. The cells were then mechanically dispersed by shaking and were filtered through a 200-μm-mesh size polyester filter (Miltenyi Biotec, Auburn, CA, USA). The cells were spun down at 2000 r.p.m. for 5 min., and the supernatant was discarded. Thereafter, the cells were plated onto sterile glass coverslips coated with murine collagen (2.5 μg/ml) in 35 mm culture dishes and cultured at 37°C in a 95% O_2_-5% CO_2_ incubator in M199 (M199 medium, 10% FBS, 2% antibiotics/antimycotics). After 24 hrs in culture, fresh medium was added.

### Flow cytometry

The samples were prepared for the determination of the percentages of lymphocytes and macrophages in the ascites fluid, as well as the primary cultured ICCs. After cell counts, 1 × 10^6^ cells were added into every tube, then the cells were rinsed with PBS and centrifuged. After 30 min. incubation with the immunofluorescence-labelled antibodies in the dark on a shaker and three washes with ice-cold staining buffer, the samples were resuspended in 500 μl of 1% paraformaldehyde and filtered through a 40-μm-mesh filter prior to sorting (this experiment was performed more than 3 times).

### Light microscopy

Observation through an inverted microscope was accomplished with a Nikon (Tokyo, Japan) inverted microscope at ×200 power. The images were captured with a conventional optical camera.

### Haematoxylin and eosin examination and immunohistochemistry of the cells in ascites fluid

After centrifugation for 10 min. at 1500 r.p.m., the cells were fixed with 4% formaldehyde and processed into a paraffin block, then sectioned. Haematoxylin and eosin staining and immunohistochemistry were then performed on the sections. The proteins of interest included CA125 (stock solution), CD68 (1:100), and CD8 (1:100).

### Immunofluorescence

The immunofluorescence expression of c-kit in ICCs was examined by fluorescence microscope. Briefly, after several days of explant culture, the culture medium was removed from the coverslips, and all of the cultured cells were fixed with 95% ethanol for 30 min. at room temperature. Prior to staining with a c-kit antibody, the samples were incubated in goat serum for 30 min. at room temperature to reduce non-specific staining. After incubation with an anti-c-kit antibody (1:50) at 4°C overnight, the samples were washed in PBS for 3 × 10 min. Then, the immunoreactivity was detected with an FITC-conjugated secondary antibody (anti-rabbit IgG, 1:100); the nuclei were labelled with DAPI. The sections were examined by fluorescence microscopy with an excitation wavelength of 488 and 594 nm.

### The morphology of the examined mice

The mice were killed after the formation of ascites. The jejunum and ileum were divided into four parts: one part for paraffin sectioning, one for frozen sectioning, one for ultrathin sectioning and a fourth section that was stored in the freezer (below −80°C). The experimental details were omitted.

### Electron microscopy

After centrifugation, the cells and the intestinal tissues were fixed for 2 hrs with 2.5% glutaraldehyde in PBS (pH 7.2). After rinsing with PBS, the tissues were post-fixed in 1% osmium tetroxide for 1 hr at 4 °C, dehydrated in a graded series of acetone, and embedded in Epon 812. Ultrathin sections were cut in slices 50–70 nm thick, double-stained with uranyl acetate and lead citrate, and examined by an electron microscope (HITACHI H-7650; HITACHI, Tokyo, Japan).

### Cell counting kit-8 assay

Briefly, ICCs were seeded at a density of 5 × 10^5^ cells/well in 96-well flat-bottomed microplates. The cells were divided into four groups: control group, Raw246.7 supernatant group (ICCs were co-cultured with Raw246.7 supernatant), ascites group (ICCs were co-cultured with ascites supernatant) and MFC supernatant group (ICCs were co-cultured with MFC supernatant). Once the cells attached to the bottom of the culture vessel, the culture medium was removed from each well. Mixtures that contained different concentrations of culture medium and supernatant solution mentioned above were added to the wells. The ratios were from 1:1 to 1:5, and the cells were cultured for 48, 72 and 96 hrs respectively. CCK8 reagent (10 μl) was added to each well of a 96-well flat-bottomed microplate that contained 100 μl of culture medium for a final concentration of 10 μl/100 μl, and incubated for an additional 4 hrs at 37°C. The absorbance rate was measured at 450 nm by an auto microplate reader (BioRad, Hercules, CA, USA). All experiments were performed in quintuplicate on three separate occasions.

### TUNEL

Apoptotic cells were evaluated using the terminal deoxynucleotidyl transferase (TDT)-mediated deoxyuridine triphosphate (dUTP) nick end labelling (TUNEL), which detected nuclear DNA fragmentation by labelling the terminal end of nucleic acids. In brief, the cells were fixed with 1% paraformaldehyde for 15 min. on ice and 70% ethanol for at least 30 min. at −20°C. They were incubated with TDT reaction buffer, TDT enzyme, and bromolated dUTP (Br-dUTP) for 60 min. at 37°C and subsequently reacted with the anti-BrdU-FITC antibody in the dark for 30 min. at room temperature. The cells were finally assessed under a fluorescence microscope; the Br-dUTP transferred to the free 3′-hydroxyl (3′-OH) groups of cleaved DNA and TDT was detected. Apoptotic cells showed condensed green fluorescence in nuclei and normal cells showed uniform green colour.

### Intestinal myoelectrical activity

The myoelectrical activity of the intestine was recorded as described below. After a 12 hrs fast, the mice were anesthetized with amobarbital sodium. A 1.5-cm midline abdominal incision was performed, and one platinum electrode was placed on the muscular layer under the serosa; the proximal electrodes were placed 1 cm beyond the initial point of the jejunum, so as not to break through the intestinal wall. With the same methods described previously, the other platinum electrode was placed at 2 cm across the length at the beginning of the jejunum. This ensured that the electrodes were located along the same axis. The following parameters were set: 200-μv voltage, 1.0-sec./div time, and 30-Hz frequency. Recordings were made for 20 min. and saved. The electrical activities of 10 mice were selected randomly and analysed using the RM6240 B/C Multi-Channel Physiological Signal Acquisition and Recorder System software, including the maximum amplitude (μv), minimum amplitude (μv), frequency and average amplitude (μv).

### Statistical analysis

SPSS 17.0 software (SPSS, Inc., Chicago, IL, USA) was used. Cell viability data were expressed with a mean ± S.E.M. and compared with the one-way anova. A *P*-value of less than 0.05 was considered statistically significant.

## Results

### The identification of the component cells in malignant ascites

**Figure 1:**
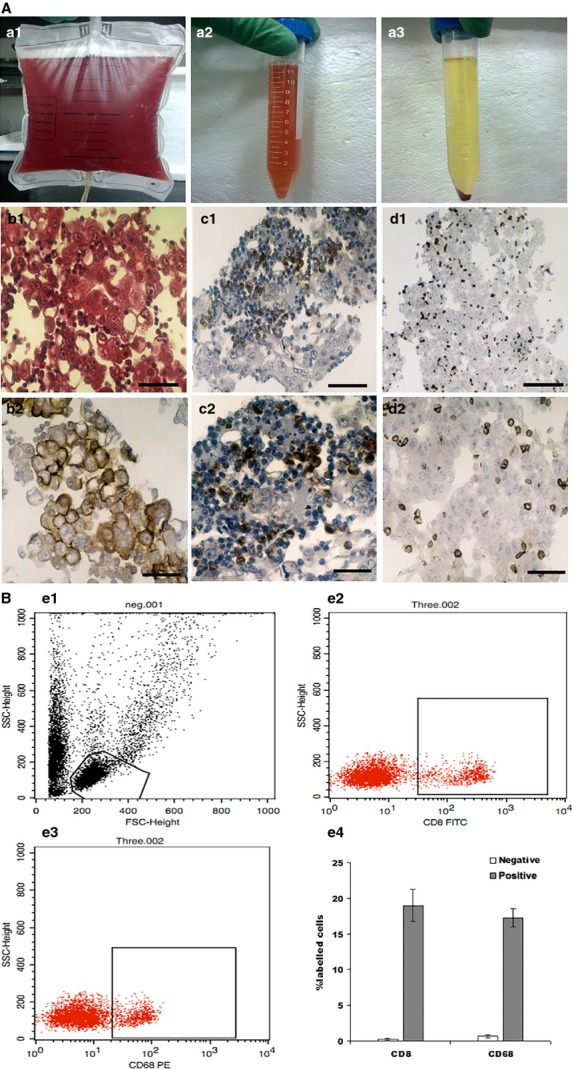
The identification of the component cells in malignant ascites. (A) The malignant ascites were obtained from patients, which were suffered from serous epithelial ovarian carcinoma (a1 and a2). The cells of ascites were showed after centrifugation (a3). The tumour cells that were obtained from malignant ascites have a large nucleus and an increased nucleosome (b1, HE (Hematoxylin-eosin staining) ×400, bar: 125 μm). Immunohistochemistry showed that CA125 was expressed in the membrane of tumour cells (b2, SP ×400, bar: 125 μm). The macrophages which expressed CD68 were distributed among the tumour cells and were detected in the cytoplasm (c1, SP ×100, bar: 500 μm and c2, SP ×400, bar: 125 μm). CD8 expression was detected in the cell membrane and CD8^+^ T lymphocytes were distributed among the cells of the ascites fluid (d1, SP ×100, bar: 500 μm and d2, SP ×400, bar: 125 μm). (B) Example of flow cytometry registration of negative group (e1), the percentage of macrophages cells were accounted for 19.06 ± 2.27% (e2), and CD8^+^ T lymphocytes were accounted for 17.26 ± 1.31% (e3) of the inflammatory cell components of malignant ascites. The account evaluation for triplicates repeated in the identical experiments (e4).

The malignant ascites and cells were obtained from the patients who were suffered from serous epithelial ovarian carcinoma (Fig.1A a1, a2, a3). Through centrifugation, the morphology was identified as follows: tumour cells are the primary cells with a large nucleus and an increased nucleosome (Fig.1A b1). Immunohistochemistry showed that CA125 was expressed in the membrane of tumour cells (Fig.1A b2). Inflammatory cells were identified in the ascites fluid, including macrophages and T lymphocytes. The macrophages that expressed CD68 were distributed among the tumour cells (Fig.1A c1, c2). Additionally, CD8^+^ T lymphocytes were distributed among the cells of the ascites fluid (Fig.1A d1, d2).

The result of the flow cytometric analysis showed that macrophages (CD68^+^ cells) were accounted for 19.06 ± 2.27% (Fig.1B e2), and CD8^+^ T lymphocytes were accounted for 17.26 ± 1.31% (Fig.1B e3) of the inflammatory cell components of malignant ascites.

### The extraction and primary culture of intestinal ICCs

**Figure 2:**
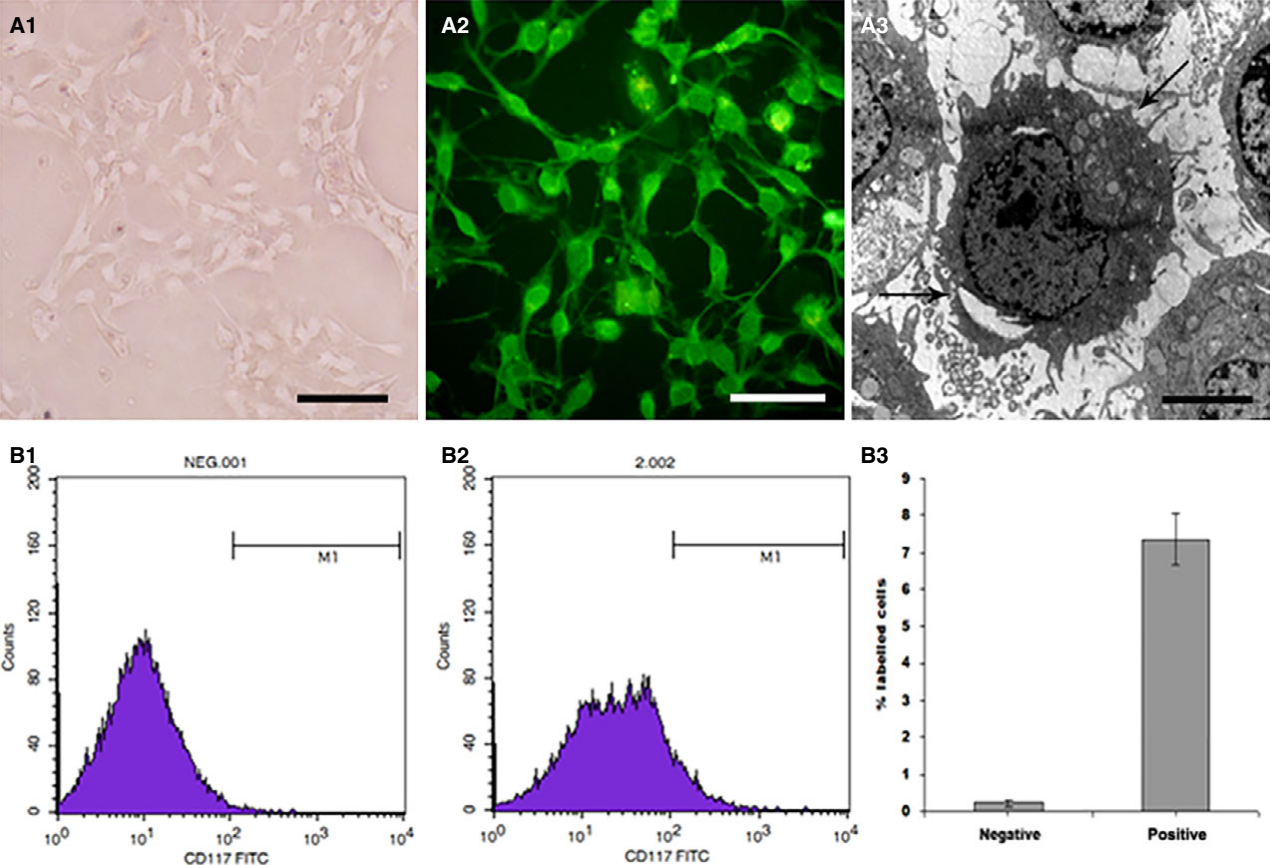
The identification of ICCs. ICCs displayed spindle morphology and obvious connections between each other under inverted microscope (A1 × 200, bar: 250 μm). Immunofluorescence demonstrated that c-kit was highly expressed in the cytoplasm and in the membrane. Single ICC showed a triangular- or stellate-shaped cell body with multiple, thin processes that extended to adjacent ICCs (A2, IF ×200, bar: 250 μm). Under the electron microscope, ICCs were observed with large prominent nuclei, numerous mitochondria and endoplasmic (A3, EM ×5000, bar: 10 μm). Example of flow cytometry registration of negative group (B1) and positive group, the percentage of ICCs reached 7.87% (B2). The account evaluation for triplicates repeated in the identical experiments (B3).

After incubation of explants in culture medium, the first cells started to grow out of the explants between 24 hrs under control conditions. Interstitial cells of Cajal cultured from the murine small intestine were identified inverted microscope, fluorescence microscope, electron microscope and flow cytometry. Under inverted microscope, ICCs displayed spindle morphology and obvious connections between each other (Fig.2 A1). Immunofluorescence demonstrated that c-kit was highly expressed in the cytoplasm and in the membrane. C-kit positive cells have a distinctive morphology that is easily recognized in cultures (Fig.2 A2). Ultrastructurally, ICCs were characterized by the presence of many mitochondria in the cytoplasm, circular or oval in shape with a large nucleus and many cytoplasmic processes with a dichotomous branching pattern (Fig.2 A3). The results of the flow cytometric analysis showed that the percentage of ICCs reached 7.87% (Fig.2 B1–B3).

### Co-culture with ICCs

**Figure 3:**
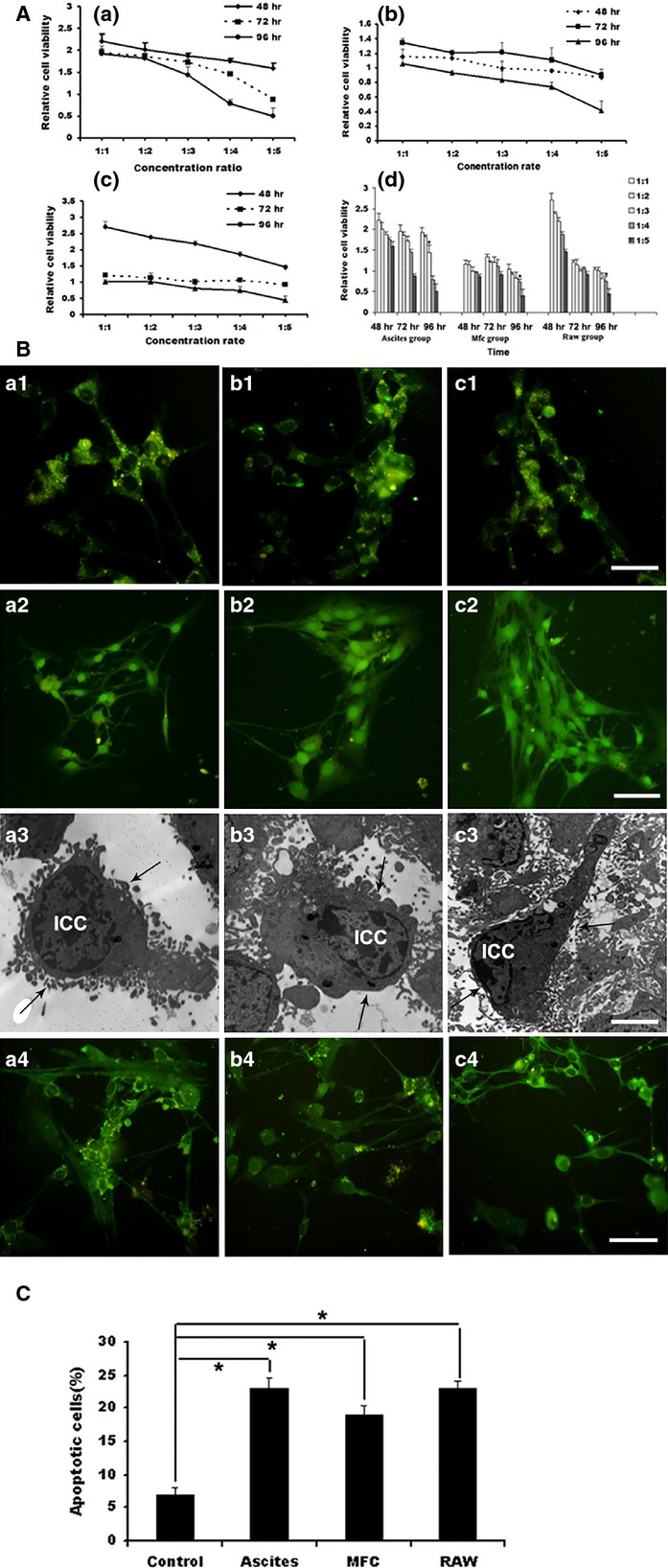
Co-cultured with ICCs. (A) The result of co-culture with ascites group, mfc and raw group by CCK8 assay. (A) ICCs were co-cultured with ascites group. (B) ICCs were co-cultured with mfc group. (C) ICCs were co-cultured with raw group. As the time extension and increased concentration of co-culture, ICCs were seriously injured. *P < 0.01, values are average of triplicate experiment and are represented as mean ± SD. (B) The expression of c-kit and TUNEL examination after co-culture with the supernatant from the ascites group (a1–a4), mfc (b1–b4) and raw group (c1–c4), ICCs were detected with immunofluorescence and electron microscopy. ICCs were damaged with volume contraction, shorten or reduced processes, aggregation of granular fluorescence in the cytoplasm and shorter or absent connections (a1, b1, c1, IF ×200, bar: 250 μm). a2, b2 and c2 was evaluated by TUNEL. Apoptotic cells showed broken nuclei with condensed green fluorescence (IF ×200, scale bar is 250 μm). Ultrastructurally, ICCs were observed with shrunken cell volume, karyopyknosis, decreased number and shorter length of processes (a3, b3, c3, black arrow, EM ×5000, bar: 10 μm). (C) The proportion of apoptotic cells was expressed as mean ± S.E.M. Asterisks, *P* < 0.05, *Significant difference between control *versus* ascites group, mfc and raw group.

As described before, ICCs were co-cultured with malignant ascites supernatant (short for ascites group), MFC supernatant (short for mfc group) and RAW246.7 supernatant (short for raw group). As the time extension and increased concentration of co-culture, ICCs were seriously injured. The optimal conditions were chosen as follows: the ratio between the normal culture volume and the volume of the ascites group was 1:3 and the appropriate co-culture time-point was 96 hrs (Fig.3A-a). The ratio between the normal culture and the volume of mfc group was 1:4 and the appropriate co-culture time-point was 96 hrs (Fig.3A-b). The ratio between the normal culture and the volume of raw group was 1:4 and the appropriate co-culture time-point was 96 hrs, as shown in Figure3A-c. The immunofluorescence expression of c-kit in ICCs was examined by fluorescence microscope. The results demonstrated that co-culture with supernatant from these three groups, ICCs were damaged in the morphology and function, such as contraction, shortened or reduced connections and increased aggregation of granular fluorescence in the cytoplasm (Fig.3B-a1, b1 and c1). Apoptosis was evaluated by TUNEL. Apoptotic cells showed broken nuclei with condensed green fluorescence, as described in Figure3B-a2, b2 and c2. Under electron microscope, ICCs demonstrated other indications of cellular injuries including shorter processes, condensed nuclei and degenerated organelles in the cytoplasm (Fig.3B-a3, b3 and c3). After treatment with SCF, the cellular injuries were ameliorated: an increased volume of the cell body, increased processes and cell connections were observed. Furthermore, a transmission electron microscope evaluation showed the decreased number of ICCs with shrunken cell volume, karyopyknosis and shorten process length (Fig.3B-a4, b4 and c4). Figure3C showed statistical analysis of TUNEL. The proportion of apoptotic cells was expressed as mean ± S.E.M. Asterisks, *P* < 0.05, *Significant difference between control *versus* ascites group, mfc and raw group.

### The impact of malignant ascites on intestinal ICCs

**Figure 4:**
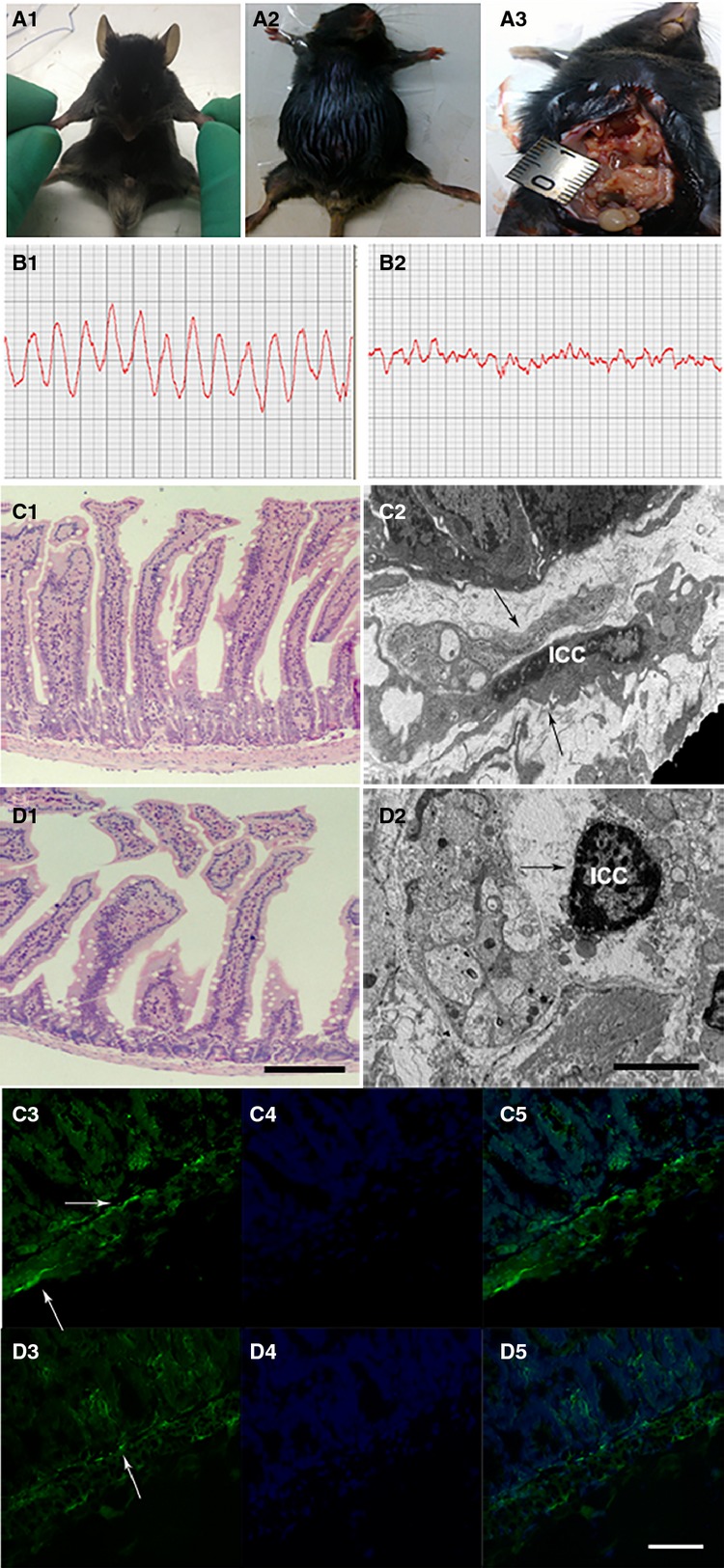
The impact of malignant ascites on intestinal ICCs. (A1) Normal C57BL/6 mouse. (A2) Animal model creation of malignant ascites. (A3) The nodules in the abdominal cavity. Control mice exhibited regular waves and stable frequencies (B1), but in mice with malignant ascites, the amplitude of the intestine was clearly reduced and irregular (B2). On light microscopy, the integrity and columnar shape of the intestinal villi were maintained; the thickness of the muscularis propria was uniform (C1, LM ×200, bar: 250 μm), in the malignant ascites group, the intestinal villi were reduced and thinner, and the muscularis propria was significantly thinner than that in the control group (D1, LM ×200, bar: 250 μm). Ultrastructurally, ICCs displayed well-centred nuclei and numerous cytoplasmic processes that stretched and connected to nerve cells in control group (C2, black arrow, EM ×4000, bar: 12.5 μm), and in the malignant ascites group, the quantity and volume of ICCs were decreased, and many vacuoles were formed in the cytoplasm in the early stage of the model group. With the progression of disease, the nuclei clearly became condensed, the cell bodies became shrunken, and the cytoplasmic processes were decreased and had no connections with other cells (D2, black arrow, EM ×4000, bar: 12.5 μm). The immunofluorescence demonstrated that c-kit was partially expressed in the muscularis layer (C3, white arrow, IF ×400, bar: 125 μm), in the malignant ascites group, immunofluorescence showed few, spotty and scattered c-kit expression in the muscularis layer (D3, white arrow, IF ×400, bar: 125 μm). (C4 and D4) Nuclei are stained blue with DAPI. (C5 and D5) merged images.

The mouse model of malignant ascites was successfully generated as shown in Figure4 A1-A3. Control mice exhibited regular waves and stable frequencies (Fig.4 B1), but in mice with malignant ascites, the amplitude of the intestine was clearly reduced and irregular (Fig.4 B2). On light microscopy, the integrity and columnar shape of the intestinal villi were maintained; the thickness of the muscularis propria was uniform (Fig.4 C1), but in the malignant ascites group, the intestinal villi were reduced and thinner, and the muscularis propria was significantly thinner than that in the control group (Fig.4 D1). Ultrastructurally, ICCs displayed well-centred nuclei and numerous cytoplasmic processes that stretched and connected to nerve cells in control group (Fig.4 C2, black arrow), and in the malignant ascites group, the quantity and volume of ICCs were decreased, and many vacuoles were formed in the cytoplasm in the early stage of the model group. With the progression of disease, the nuclei clearly became condensed, the cell bodies became shrunken, and the cytoplasmic processes were decreased and had no connections with other cells (Fig.4 D2, black arrow). The immunofluorescence demonstrated that c-kit was partially expressed in the muscularis layer (Fig.4 C3). In the malignant ascites group, immunofluorescence showed few, spotty and scattered c-kit expression in the muscularis layer (Fig.4 D3).

## Discussion

Malignant ascites is one of the dangerous complications that can occur in the latter stage of abdominal malignant tumours, such as ovarian cancer, primary liver cancer and gastric cancer 14,15. The patients who are diagnosed with cancer and malignant ascites often experience symptoms of intestinal peristalsis disorder such as abdominal pain and abdominal distension. In this study, we demonstrated that the number of intestinal ICCs were significant decreased, the morphology and function of ICCs were damaged when simulated TME *in vitro*. Furthermore, using the mice model, we illustrated that intestinal activity was damaged with morphological changes of ICCs under the condition of malignant ascites.

In general, tumour cells interact with other cells in the TME, where they regulate gene expression in those cells to affect the phenotype. During this interaction, many inflammatory cells, cytokines and chemokines are produced and form a complicated network. Moreover, the cells in this network could directly affect the host cells and transform the surrounding environment by endocrine, exocrine and paracrine means 1,2. Meanwhile, these changes could damage some of the functional cells, such as intestinal ICCs 3,4. Interstitial cells of Cajal in the myenteric plexus are pacemaker cells, which generate characteristic slow, spontaneous waves. They mediate inputs from the enteric nervous system to smooth muscles and establish the smooth muscle membrane potential gradient 16,17. However, they are also sensitive to harmful factors. It has been reported that a reduced number and pathological changes of ICCs may lead to the injuries of intestinal motility 5,6.

A study of the tumour micromatrix found that the number of lymphocytes and macrophages was notably increased compared to normal tissues 18. Our research demonstrated that tumour cells, lymphocytes and macrophages are the primary cellular components of malignant ascites. Previous study reported that the initial population of TILs are mainly composed of CD3^+^ T cells, including CD4^+^ T lymphocytes and CD8^+^ T lymphocytes. Tumour-infiltrating lymphocytes could kill tumour cells and induce apoptosis of these cells because of the higher rate of CD8/Fas-positivity cells 7–9. In this study, we found that lymphocytes that were rich in malignant ascites, suggesting these T cells may not only induce apoptosis of tumour cells, but also cause the contraction and apoptosis of intestinal ICCs.

The TME contains many TAMs that originate from peripheral blood mononuclear cells after tumour cells metastasize to the abdominal cavity. Under pathological circumstances, TAMs could damage normal cells, participate in tissue reconstruction, promote infiltration of inflammatory cells and induce the immune response 19–21. It is believed that the TME is a complex network that could produce cytokines, inflammatory mediators and inflammatory cells, which is also called the inflammatory microenvironment 22–24. Therefore, the TME may have a negative effect on intestine and intestinal ICCs through inflammatory network.

Our data also demonstrated that TILs and TAMs in ascites fluid could damage the intestinal ICCs. The functions of ICCs were partly repaired after administrating an appropriate amount of SCF. Interstitial cells of Cajal express c-kit, and SCF, a multi-functional cellular factor, is the ligand of c-kit. The SCF/c-kit pathway plays an important role in the phenotypic maintenance, proliferation, differentiation and regulation of ICCs and intestinal motility 25,26. Abnormal SCF/c-kit signalling is the main cause of reduced ICCs and the disappearance of slow waves. Based on the above principle and a series of studies, the damage or loss of gastrointestinal ICCs could lead to abnormal gastrointestinal peristalsis 27–29.

In conclusion, our data suggested that malignant ascites may damage ICCs of the small intestine and lead to intestinal motility disorders. Inflammatory cells, such as, TILs and TAMs in ascites, may be the main cause of ICCs injury and dysfunction of intestine.
